# Lipoxins, RevD1 and 9, 13 HODE as the most important derivatives after an early incident of ischemic stroke

**DOI:** 10.1038/s41598-020-69831-0

**Published:** 2020-07-30

**Authors:** Małgorzata Szczuko, Dariusz Kotlęga, Joanna Palma, Agnieszka Zembroń-Łacny, Anna Tylutka, Monika Gołąb-Janowska, Arleta Drozd

**Affiliations:** 1 Department of Human Nutrition and Metabolomics, Pomeranian Medical University, Broniewskiego 24 street, 71-460 Szczecin, Poland; 2 Department of Neurology, Pomeranian Medical University, Szczecin, Poland; 3 Department of Neurology, District Hospital, Głogów, Poland; 4 Department of Applied and Clinical Physiology, University of Zielona Góra, Zielona Gora, Poland

**Keywords:** Cardiology, Diseases, Medical research, Neurology

## Abstract

There is limited information available regarding the association of plasma free fatty acids (FFA) and inflammation mediators with ischemic stroke. At the same time, new treatment strategies are being pursued. The aim of this study was to carry out a thorough analysis of inflammation with multiple FFA-derivative mediators after and ischemic stroke and standard treatment. HPLC separations of 17 eicosanoids were performed using an Agilent Technologies 1,260 liquid chromatograph. The profiles of the esters of fatty acids were labelled by means of gas chromatography. FFA, and eicosanoid profiles in the group of patients after ischemic stroke significantly differed from the profile of the control group. Studies confirmed the involvement of derivative synthesis pathways responsible for the inflammation, especially palmitic acid (9 and 13 HODE), arachidonic acid, EPA and DHA. Arachidonic acid derivatives were synthesised on 5LOX, 15 LOX and COX pathways with the participation of prostaglandins while omega 3 derivatives strengthened the synthesis of resolvins, RevD1 in particular. The ability to accelerate the quenching of inflammation after ischemic stroke seems to be a promising strategy of stroke treatment in its early stage. In this context, our study points to lipoxins, RevD1, and 9, 13 HODE as the most important derivatives.

## Introduction

Stroke is one of the main causes of death and disability throughout the world^1^. There are two main types of stroke, ischemic and hemorrhagic stroke, which constitute 80–85% and 15–20% of all strokes, respectively^2,3^. Ischemic stroke concerns both old patients and young adults^4^. In old patients, it is associated with the progression of atherosclerosis and the frequent occurrence of atrial fibrillation (AF), whereas mechanisms other than atherosclerotic, including patent foramen ovale and mitral valve prolapse, may contribute to the pathogenesis of stroke in young patients^5^. In terms of epidemiology, stroke is one of the most sex-dependent illnesses. Women have a lower index of ischemic stroke than men, even after menopause, regardless of the country of origin or ethnicity^6,7^. However, the results of many clinical trials are worse in older women than men^8^. The biological basis of this sexual dimorphism is fascinating due to the involvement of conditions that depend on hormones, endogenic oestradiol and progesterone, which are both vasoprotective and neuroprotective^9,10^.

**Figure 1:**
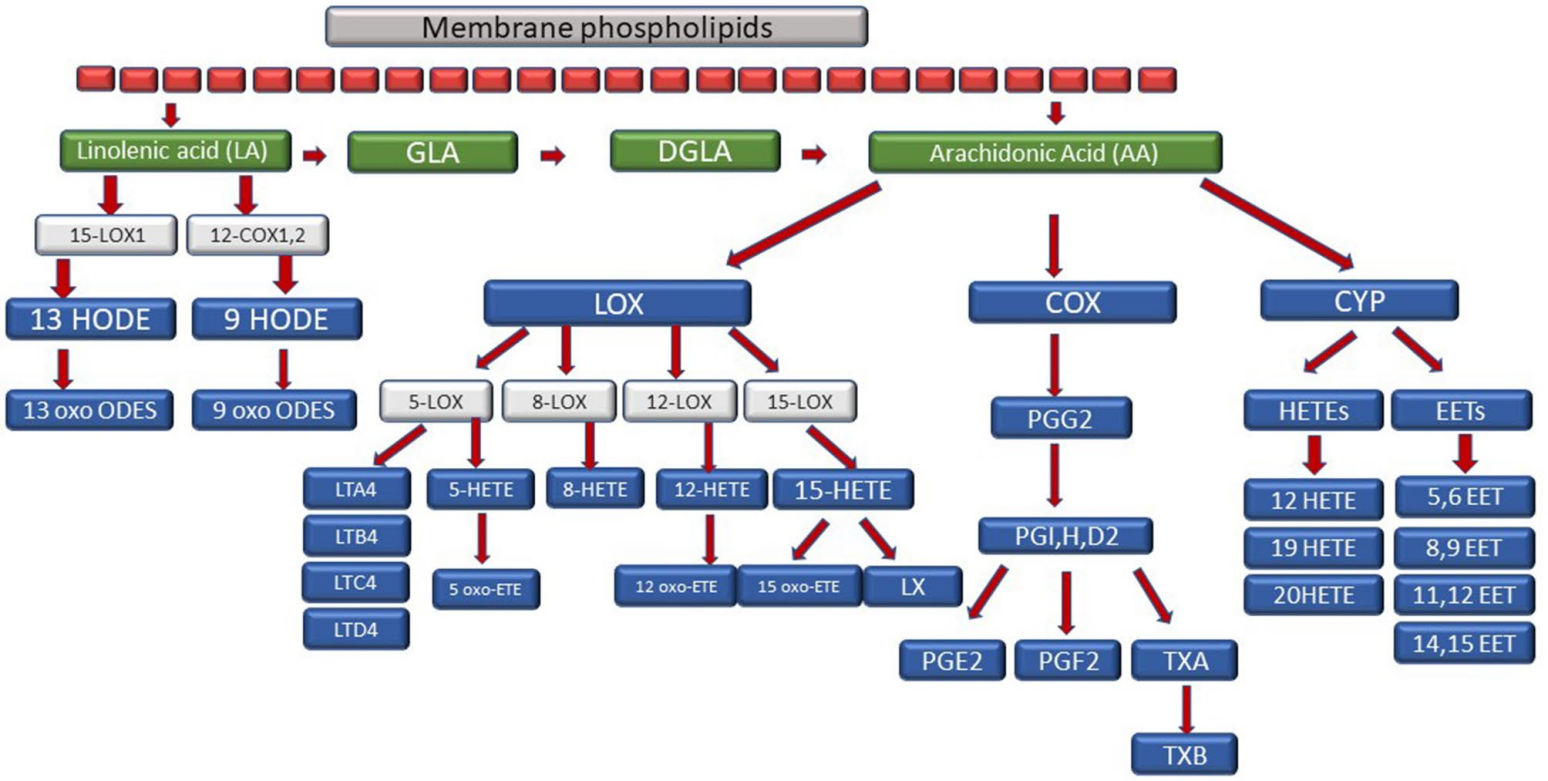
Synthesis of inflammation mediators, plasma free fatty acid (FFA) derivatives.

Epidemiological and clinical studies confirmed that food enriched with eicosapentaenoic acid (EPA), docosahexaenoic acid (DHA) and fish oil decreases the occurrence of cardiovascular diseases and stroke^11^. However, the role of n-6 poly-unsaturated fatty acids (PUFA) and the proportions of n-3/n-6 are a controversial subject^12^. Higher intake of linoleic acid (LA) and higher levels of arachidonic acid (AA) in circulation are dangerous^13,14^. The proposed damage mechanism is based on increased inflammation, driven by the conversion of AA into proinflammatory and prothrombotic eicosanoids, such as prostaglandin E2, thromboxane A2 and leukotriene B4^15^. There remains limited information on the association between plasma free fatty acids (FFA), inflammation mediators and ischemic stroke. This work is one of the first studies describing the level of inflammation mediators in such detail, but the process was not fully elucidated. Our observations contribute to the understanding of the processes that accompany stroke and help improve treatment. Both ischemic and hemorrhagic strokes are associated with severe functional disability and high mortality^16^. The limitation of the functional consequences of a stroke may help patients recover faster and limit the costs of long-term treatment and rehabilitation. So far, a number of publications have focused on the connection between the risk of cardiovascular illnesses/mortality and FFA, focusing on docosahexaenoic acid (DHA), linoleic acid, arachidonic acid and palmitic acid, but the results are unclear^17–21^. A study by Harris et al. did not show any connection between the level of linoleic acid and mortality, whereas the research conducted by De Goede et al. revealed no connection with stroke^18,21^. However, higher levels of linoleic acid were associated with a decreased risk of stroke in general in the population of Swedish men^22^, and of ischemic stroke in the ARIC study^23^. Similar connections referring to linoleic acid were observed in Asian populations^24,25^. When evaluating the influence of palmitic acid, it was determined that the circulating palmitic acid is associated with the increased risk of ischemic stroke in women after menopause^26^ and with the increased risk of a stroke in general in men^22^. In ARIC, although the levels of saturated fatty acids (SFA) in general were associated with the 64% increased risk of stroke, the results were not significant for palmitic acid itself^23^. Other studies did not show a connection between palmitic acid and ischemic heart disease^17^. The differences in the interpretation of the connection between FFA (palmitic acid in particular) and stroke could have resulted from a number of factors, including the timing of the study after the stroke and not accounting for the fact that individual acids are the precursors of mediators of inflammation whose synthesis could have been amplified and whose use could have been elevated in the inflammatory response. Therefore, higher use of FFA for the processes of synthesis of derivative compounds could have obscured the influence of individual acids on the occurrence of stroke. This is why we decided to study FFA and their derivatives. Elaidic acid (18: 1n9) and arachidonic acid (20: 4n6) were positively correlated, whereas lignoceric acid (24: 0) had a negative correlation with cardioembolic (CE) stroke in the Korean population^27^. No significant difference was observed in relation to EPA/AA and DHA/AA between three subgroups of strokes (hemorrhagic, ischemic strokes and lacunar) but the DGLA/AA relation was much higher in patients with lacunar infarction (LI) than in CE patients^28^. The high ratio of EPA/AA was associated with a good prognosis in ischemic stroke, suggesting that nutritional habits before the stroke influence the exacerbation and course of ischemic stroke in patients^28^. Moreover, it has been established that the proportions of EPA/AA and DHA/AA may be specific markers for younger patients after a stroke. The EPA/AA ratio may be associated with the calcification of the aortic arch in older stroke patients, with numerous infarctions and with the disease of white matter in middle-aged stroke patients^29^. For a better understanding of the cascade of FFA transformation into inflammatory mediators, a diagram is shown in Fig. 1.

Acetylsalicylic acid (aspirin) and statins are the basic drugs for the secondary prevention of stroke^30^. They also have an influence on the eicosanoids. Aspirin is the permanent inhibitor of COX-1. Moreover, both aspirin and statins can modify products of COX-2 activity, such as 15-HETE, HEPE and 17(H)DHA, which are precursors of specialized pro-resolving mediators^31^ Antiplatelet therapy is the basis for the secondary prevention of strokes^32^. No response to antiplatelet treatment is associated with worse clinical results^33^. The average annual risk of a future ischemic stroke after an initial ischemic stroke/TIA (transient ischemic attack) is from 3 to 4%^34^. Some scientists interpret such individual differences in relation to antiplatelet drugs as “aspirin/clopidogrel resistance”^35–37^. The aim of this study was to thoroughly analyze inflammation with multiple FFA derivative mediators after ischemic stroke and standard treatment.

## Materials and methods

### Patients and sample collection

For the purpose of this study, we received the approval of the Bioethics Committee at the Regional Medical Council in Zielona Góra, Poland No 08/73/2017. Written informed consent was obtained from all subjects.

The study group consisted of 75 patients of Caucasian origin with ischemic stroke (IS) and 35 potentially healthy patients constituting the control group (CG). Patients with ischemic stroke were hospitalized at the Department of Neurology of the District Hospital in western Poland, whereas the control group consisted of participants of University of the Third Age in western Poland. The inclusion criteria included a diagnosis of ischemic stroke basing on symptoms, computer tomography (TK) of the brain and/or brain nuclear magnetic resonance (NMR), and a conscious consent to participate in the study. Computer tomography was performed by means of Somatom Emotion 16 slices (Siemens 2015, Munich, Germany), and for brain NMR a Magnetom Essenza 1,5 T (Siemens 2017, Munich, Germany) was used. The exclusion criteria included speech and/or consciousness disorders that would make it impossible to receive conscious consent to participate in the study, and the presence of an active infection or a neoplastic disease. Fasting blood was collected from study group patients on the seventh day after the incident. Blood samples for chromatographic determinations were collected from patients into 10 ml polypropylene tubes containing EDTA. Then, the blood samples were centrifuged at 3,000 rpm for 10 min using a refrigerated centrifuge. The separated plasma was collected into Eppendorf tubes and stored at − 80 °C until analysis. Standard treatment was conducted in accordance with the up-to-date recommendations for the management of acute stroke, based on the guidelines of the Expert Group of the Section of Cerebrovascular Diseases of the Polish Neurological Association^38^ and guidelines for the prevention of stroke in patients with stroke and transient ischemic attack from the American Heart Association/American Stroke Association^39^. Only five patients with IS used sporadic fish oil or omega-3 capsules. The description of both groups and the participation of sexes is presented in Table 1.

**Table 1:** Summary of patients after ischemic stroke (IS) and the control group (CG).

Parameter	IS mean ± SD	CG mean ± SD	p
Age (years)	66.08 ± 11.9	71.31 ± 5.42	NS
Height (cm)	167.85 ± 8.40	165.26 ± 8.54	NS
Body mass (kg)	80.98 ± 15.33	74.05 ± 12.92	NS
BMI (kg/m^2^)	28.8 ± 5.01	26.98 ± 3.34	NS
Number of women	42	18	–
Number of men	33	17	–

*NS* not significant.

### Chemicals and reagents

All used reagents were GC or HPLC grade. Chloroform (cat no. 34854), methanol (cat no. 1.06018), hexane (cat no. 270504), acetonitrile (cat no. 20060.320), acetic acid (cat no. 45754), boron trifluoride in methanol (cat no. B1252), NaCl (cat no. S3014) and 2,6-Di-tert-butyl-4-methylphenol (BHT) (cat no. B1378), ethyl acetate (cat no. 34858), and hydrochloric acid (cat no. H1758) were purchased from Merck KGaA (Warsaw,Poland). Double-distilled water was obtained from a Milli-Q Water System (Millipore, Billerica, MA, USA). Buffers used for HPLC analysis were filtered through 0.22 µm nylon filters (Agilent).

### FAME extraction

Fatty acids (FAs) were extracted based on the protocol published by Folch^40^ with minor modifications^41^. For serum samples, 0.5 ml serum was added to a 2.5 ml of Folch mixture (chloroform: methanol: v/v 2:1) and 100 μl of BHT and 100 μl of internal standard C21:0. This was mixed for 20 min using an incubator shaker (New Brunswick Scientific, Excella E24 Series). Samples were then centrifuged at 15,000 rpm for 15 min. (Eppendorf, Centrifuge 5804R). Serum (1 ml) was saponified with 1 ml of 2 M KOH methanol solution at 70 °C for 20 min and then methylated with 2 ml of a 14% solution of boron trifluoride in methanol (Sigma-Aldrich, Germany) under the same conditions. Then, 2 ml n-hexane and 10 ml of saturated NaCl solution were added. After completely separating the upper (n-hexane phase) and lower layers, the n-hexane phase (1 ml) was collected.

### Gas chromatography (GC) analysis

GC was performed using an Agilent Technologies 7890A GC System equipped with a SUPELCOWAX 10 Capillary GC Column (L × I.D. 15 m × 0.10 mm, df 0.10 μm; Supelco, cat no. 24343). The temperature was held at 40 °C for 0.5 min, and was then increased by 25 °C/min up to 195 °C for 0 min, then by 3 °C/min to 205 °C for 0 min, and by 8 °C/min to 250 °C for 0.5 min. The total analysis time was 16.158 min and the gas flow rate was 1 ml/min, with nitrogen as the carrier gas.

**Figure 2:**
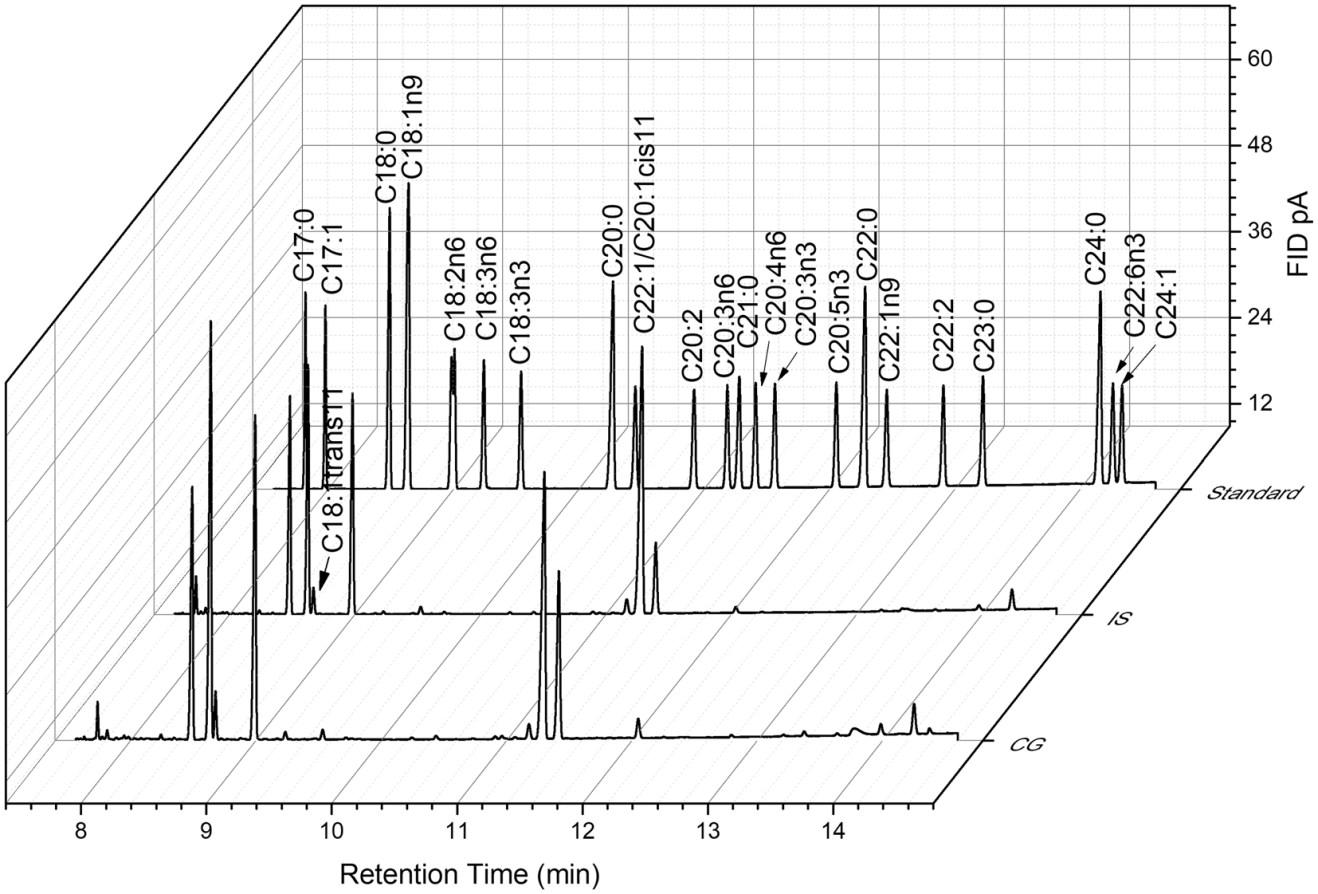
Representative GC chromatogram of some statistically significant fatty acids for ischemic stroke (IS) and control group (CG).

FAs were identified by comparing their retention times with those of Food Industry FAME Mix (cat. no 35077) (Restek), supplemented with the following single standards: C18:4 (stearidonic acid methyl ester, cat. no 10005000,), C22:4n6 (docosatetraenoic acid methyl ester, cat. no 10006866) and C22:5n3 (docosapentaenoic acid methyl ester, cat. no 21124, Cayman Chemical) by using ChemStation Software (Agilent Technologies, Cheadle, UK). To control the fatty acid retention times, C21:0 (heneicosanoic acid, cat. no H5149, Merck KGaA) was used as the internal standard. Results are presented as the percentage of the individual fatty acids in the total mass of fatty acids from the examined samples (Fig. 2).

### Eicosanoids extraction

The following were extracted from the plasma by using solid-phase extraction RP-18 SPE columns (Agilent Technologies, UK): 5(S),6(R)-Lipoxin A_4_ (cat no. 20110), 5(S),6(R),15(R)-Lipoxin A_4_, (cat no. 20110), 5-HETE, (cat no. 34230), 5-oxoETE (cat no. 34250), 12-HETE (cat no. 34570), 15-HETE, (cat no. 34720), 16(R)/16(S)-HETE (cat no. 10004385/10004384), 9-HODE (cat no. 38410), 13-HODE, (cat no. 38610), 18-HEPE (cat no. 3284), 17-HDHA (cat. no 3365), 10(S)17(R)DiDHA (Protectin DX) (cat no. 10008128), Maresine1 (cat no. 10878), Leukotriene B4, (cat no. 20110), Prostaglandin E2 (cat no. 14010), Prostaglandin B2 (cat no. 11210), and Rev D1 (cat. no 10012554), Rev E1 (cat no. 10007848). For the extraction of eicosanoids, 0.5 ml plasma was added to 1 ml acetonitrile for protein precipitation and 50 µl of internal standard (1 µg/ml). After 15 min of incubation at − 20 °C, samples were centrifuged at 10,000 rpm for 10 min using a chilled centrifuge (Eppendorf, Centrifuge 5804R). Supernatants were transferred to new collection tubes and 4.5 ml of 1 mM HCl was added. The pH of each sample was adjusted to 3 by adding 30–50 µl of 1 M HCl. The columns were activated with consecutive washes of 3 ml 100% acetonitrile and 3 ml 20% acetonitrile in water. The samples were loaded and double washed with 3 ml 20% acetonitrile in water. Eicosanoids were then eluted with 1.5 ml of a mixture of methanol and ethyl acetate (1/1 v/v), dried under a vacuum and dissolved in 100 µl of 60% methanol in water with 0.1% acetic acids. Samples were immediately analyzed using HPLC.

### HPLC operating parameters

**Figure 3:**
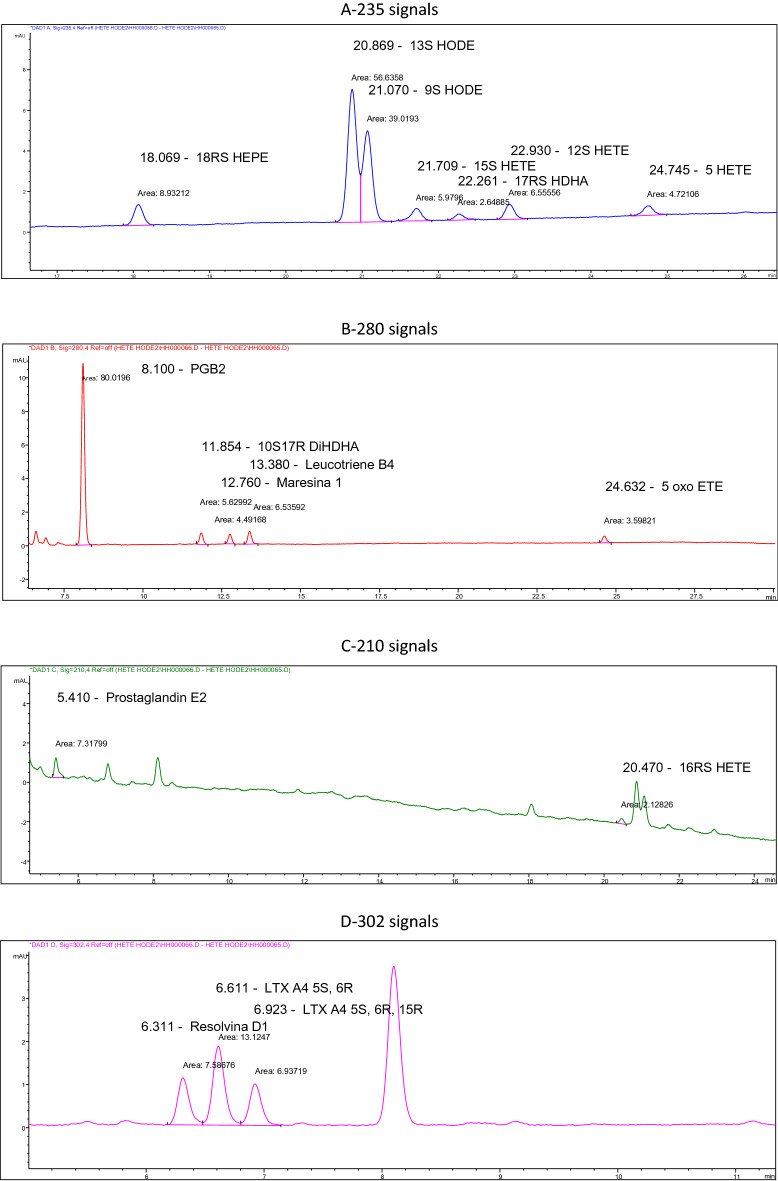
Analyzed patterns of inflammatory mediators (A-235; B-280; C-210; D-302 signals).

The HPLC separations were performed using an Agilent Technologies 1,260 liquid chromatograph, consisting of a degasser (model G1379B), bin pump (model G1312B), column oven (model G1316A) and diode array detector (model G1315CDAD VL+). Samples were injected using G1329B. An Agilent ChemStation software (Agilent Technologies, Cheadle, UK) was used for instrument control, data acquisition and analysis. The separation was completed using a Thermo Scientific Hypersil BDS C18 column (100 × 4.6 mm 2.4 µm; cat no. 28102–154630). The temperature of the column oven was set to 20 °C. The results of the FFA level expressed in ug/mL were converted into the share of the FFA percentage in plasma, as recommended by other authors^42,43^. A gradient method was used, where the mobile phase was composed of a mixture of solvent A (methanol/water/acetic acid, 50/50/0.1, v/v/v) and B (methanol/water/acetic acid, 100/0/0.1, v/v/v). The content of buffer B in the mobile phase was 30% at 0 to 2 min of separation, increased linearly to 80% at 33 min, was 98% between 33.1 and 37.5 min, and was 30% between 40.3 and 45 min. The flow rate was 1.0 ml/min. The sample injection volume was 60 ul. The DAD detector monitored peaks by adsorption at 235 nm (Fig. 3A) for 16-HEPE, 17-HDHA, 9-HODE, 13-HODE, 5-HETE, 12-HETE and 15-HETE, at 280 nm (Fig. 3B) for PGB2 (Prostaglandin B_2_, internal standard) and 5oxoETE, Rev E1, Protectin DX, Maresine1, Leukotriene B4 at 210 nm (Fig. 3C) for Prostaglandin E2, 16-HETE and 16-HETE (the latter two were eluted as one peak) and at 302 nm (Fig. 3D) for 5(S),6(R)-Lipoxin A4, 5(S),6(R), 15(R)-Lipoxin A4 and RevD1. Absorbance spectra of peaks were analyzed to confirm the identification of analytes. The quantification was based on peak areas with internal standard calibration. Quantitative analysis was conducted using ChemStation Software (Agilent Technologies, Cheadle, UK)^44,45^.

### Statistical analysis

Statistical analyses were performed with Statistica 12 (Statsoft, Kraków, Poland). Parametric tests were used because the distribution in most cases was normal (Shapiro–Wilk test). Non-parametric Mann–Whitney tests for comparisons between groups (ischemic stroke and control group) in which *p* < 0.05 was considered statistically significant. We performed a post-hoc power analysis using G power software (Dusseldorf, Germany). The power of tests which showed statistical significance was above the recommended level of 0.8, with the exception of arachidonic acid (power 0.64) and 5 oxo ETE (power 0.74).

## Results

The profile of FFA in the group of patients after ischemic stroke (IS) differed significantly from the profile of the control group (CG), as presented in Table 2. The IS group exhibited significantly higher levels of C16:0 palmitic acid, C16:1 palmitoleic acid, C18:1n9 ct oleic acid, C18:1 vaccinic acid, C18:3n6 gamma linoleic acid, C20:0 arachidic acid, C22:0 behenic acid, C22:1cis13, C24:0 lignoceric acid, and C24:1 nervonic acid.

**Table 2:** Comparison of plasma free fatty acid (FFA) content in the plasma between the ischemic stroke (IS) and control (CG) groups (%).

FFA [%]	Ischemic stroke IS mean ± SD	Control group CG mean ± SD	p-value
C13:0 tridecanoic acid	0.307 ± 0.092	0.378 ± 0.120	**0.0007**
C14:0 myristic acid	1.208 ± 0.381	1.138 ± 0.353	0.3403
C14:1 myristolenic acid	0.070 ± 0.038	0.071 ± 0.041	0.8256
C15:0 pentadecanoid acid	0.217 ± 0.108	0.211 ± 0.054	0.7521
C15:1 cis-10-pentadecanoid acid	0.081 ± 0.036	0.135 ± 0.040	**0.0000**
C16:0 palmitic acid	26.802 ± 1.742	25.930 ± 1.451	**0.0094**
C16:1 palmitoleic acid	2.134 ± 0.747	1.733 ± 0.634	**0.0048**
C17:0 heptadecanoic acid	0.302 ± 0.050	0.330 ± 0.049	**0.0051**
C17:1 cis-10-heptadecanoid acid	0.091 ± 0.035	0.132 ± 0.037	**0.0000**
C18:0 stearic acid	13.314 ± 1.983	14.833 ± 2.144	**0.0003**
C18:1n9 ct oleic acid	22.591 ± 3.713	18.454 ± 2.737	**0.0000**
C18:1 vaccinic acid	1.978 ± 0.351	1.711 ± 0.278	**0.0001**
C18:2n6c linoleic acid	11.538 ± 2.333	12.681 ± 1.828	**0.0093**
C18:2n6t linoleic acid	6.141 ± 1.931	7.485 ± 1.446	**0.0002**
C18:3n6 gamma linoleic acid	0.386 ± 0.192	0.252 ± 0.083	**0.0001**
C18:3n3 linolenic acid	0.504 ± 0.159	0.775 ± 0.350	**0.0000**
C18:4 stearidonic acid	0.057 ± 0.027	0.052 ± 0.019	0.3029
C20:0 arachidic acid	0.206 ± 0.073	0.148 ± 0.040	**0.0000**
C22:1/C20:1 cis11-eicosanic acid	0.179 ± 0.069	0.186 ± 0.059	0.5433
C20:2 cis-11-eicodienoic acid	0.151 ± 0.034	0.175 ± 0.031	0.0002
C20:3n6 eicosatrienoic acid	1.283 ± 0.309	1.246 ± 0.232	0.5167
C20:4n6 arachidonic acid	6.305 ± 1.314	6.898 ± 1.511	0.0323
C20:3n3 cis-11-eicosatrienoic acid	0.031 ± 0.014	0.048 ± 0.011	**0.0000**
C20:5n3 EPA	0.603 ± 0.258	1.139 ± 0.745	**0.0000**
C22:0 behenic acid	0.225 ± 0.098	0.087 ± 0.049	**0.0000**
C22:1n9 13 erucic acid	0.037 ± 0.016	0.034 ± 0.016	0.3982
C22:2 cis-docodienoic acid	0.017 ± 0.011	0.024 ± 0.014	**0.0023**
C23:0 tricosanoic acid	0.233 ± 0.152	0.255 ± 0.431	0.6944
C22:4n6 docosatetraenoic acid	0.223 ± 0.116	0.279 ± 0.149	**0.0282**
C22:5w3 docosapentaenoic acid	0.460 ± 0.231	0.572 ± 0.097	**0.0053**
C24:0 lignoceric acid	0.153 ± 0.076	0.044 ± 0.041	**0.0000**
C22:6n3 DHA	1.752 ± 0.531	2.381 ± 0.708	**0.0000**
C24:1 nervonic acid	0.400 ± 0.247	0.109 ± 0.228	**0.0000**

Text in bold (p-value): statistically significant; p values are 0.0000: statistically significant.

In comparison to the control group, statistically lower levels were observed in the IS group for for C13:0 tridecanoic acid, C15:1 cis-10-pentadecanoid, C17:0 heptadecanoic acid, C17:1 cis-10-heptadecanoid acid, C18:0 stearic acid, C18:2n6c linoleic acid, C18:2n6t linoleic acid, C18:3n3 linolenic acid, C20:2 cis-11-eicodienoic acid, C20:3n3 cis-11-eicosatrienoic acid, C20:5n3 EPA, C22:2 cis-docodienoic acid, C22:4n6 docosatetraenoic acid, C22:5w3 docosapentaenoic acid, and C22:6n3 DHA (Table 2).

**Figure 4:**
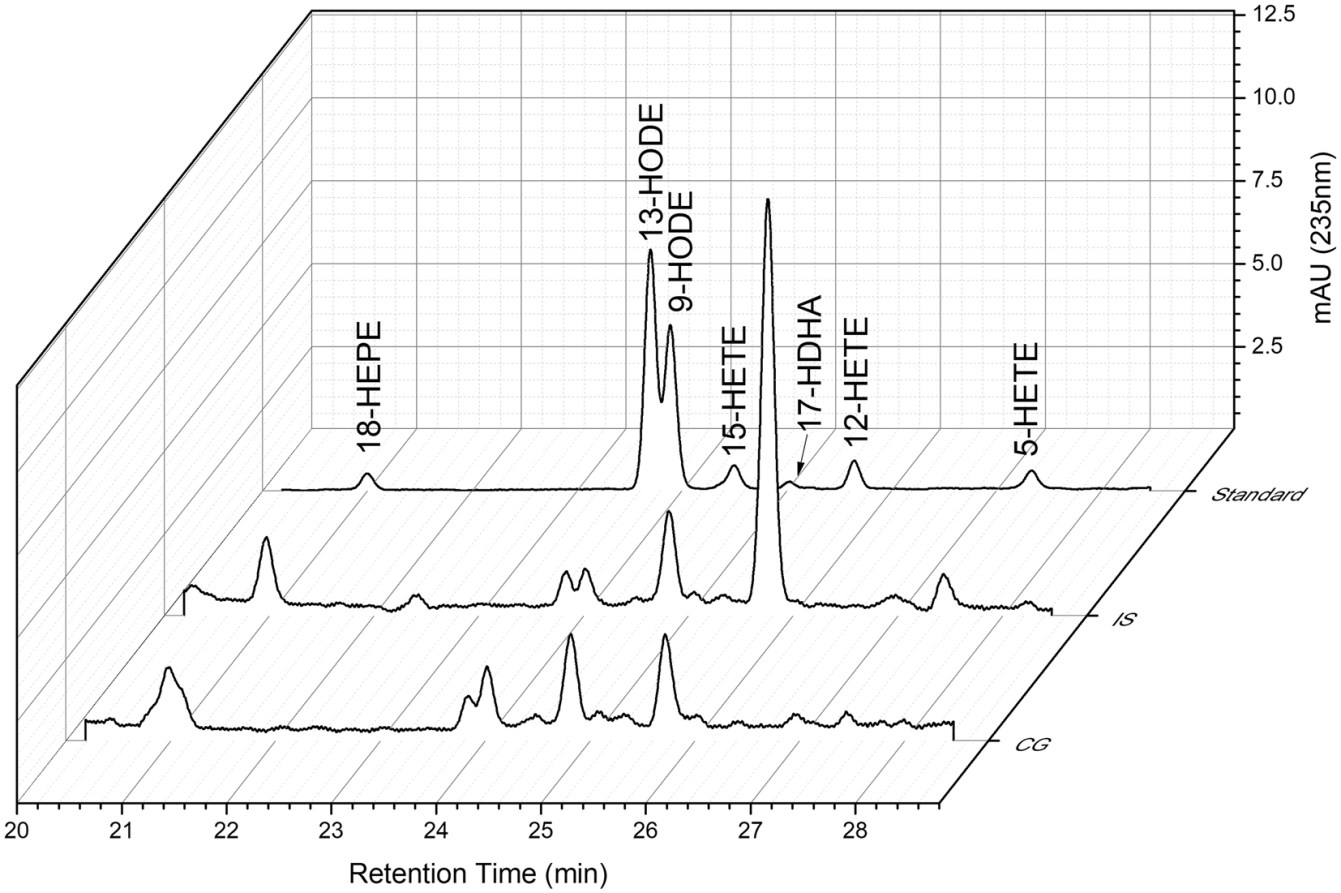
Representative HPLC chromatogram of statistically significant eicosanoids for ischemic stroke (IS) and control group (CG).

**Figure 5:**
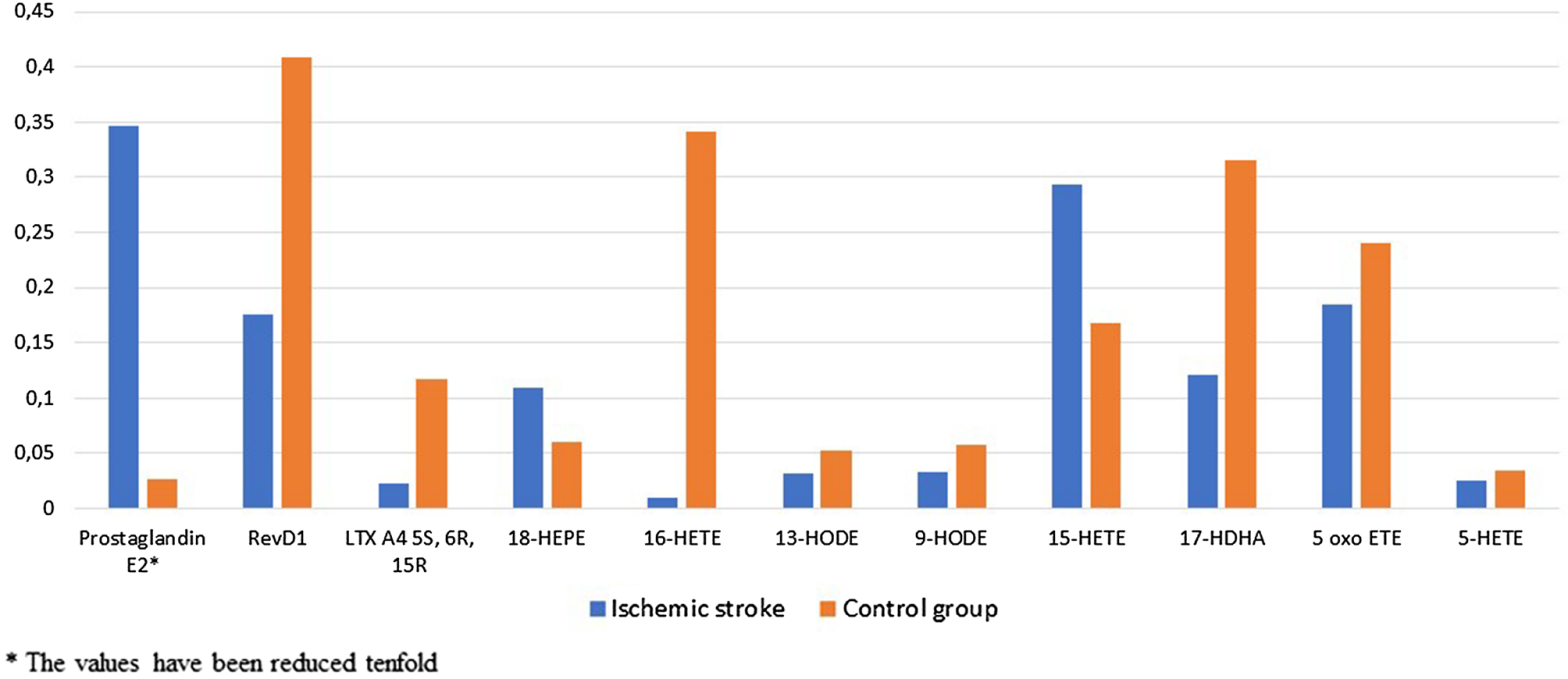
Statistically significant differences in the level of inflammatory mediators (ug/ml).

When analyzing the level of individual mediators that are FFA derivatives, significant differences between the studied groups (IS and CG) were observed, as shown in Table 3, Figs. 4 and 5.

**Table 3:** Comparison of average levels of mediators in the plasma between ischemic stroke (IS) and control group (CG) patients (ug/ml).

Inflammation mediators	Ischemic stroke IS mean ± SD	Control group CG mean ± SD	p-value
RevE1	0.061 ± 0.094	0.057 ± 0.055	0.7867
Prostaglandin E2	3.464 ± 4.463	0.261 ± 1.275	**0.0001**
RevD1	0.176 ± 0.255	0.409 ± 0.544	**0.0032**
LTX A4 5S, 6R	0.017 ± 0.147	0.004 ± 0.015	0.6173
LTX A4 5S, 6R, 15R	0.023 ± 0.042	0.117 ± 0.055	**0.0000**
Protectin DX	0.047 ± 0.064	0.057 ± 0.071	0.4442
MaR 1	0.032 ± 0.016	0.031 ± 0.007	0.7634
Leukotriene B4	0.027 ± 0.014	0.025 ± 0.008	0.4838
18-HEPE	0.109 ± 0.037	0.060 ± 0.026	**0.0000**
16-HETE	0.010 ± 0.067	0.341 ± 0.278	**0.0000**
13-HODE	0.032 ± 0.029	0.053 ± 0.020	**0.0003**
9-HODE	0.033 ± 0.028	0.058 ± 0.057	**0.0023**
15-HETE	0.293 ± 0.203	0.168 ± 0.098	**0.0015**
17-HDHA	0.121 ± 0.085	0.316 ± 0.374	**0.0000**
12-HETE	1.774 ± 1.127	1.783 ± 1.585	0.9713
5 oxo ETE	0.184 ± 0.102	0.240 ± 0.127	**0.0152**
5-HETE	0.025 ± 0.013	0.034 ± 0.007	**0.0001**

Text in bold (p-value): statistically significant; p values are 0.0000: statistically significant.

Significantly higher inflammatory mediator levels were observed in the IS group for Prostaglandin E2, 18RS HEPE, 15S HETE, whereas in the case of LTX A4 5S(6R) 15R, 16RS HETE, 13S HODE, 9S HODE, 17RS HDHA, 5 oxo ETE, 5 HETE and RevD1 the levels were significantly lower in comparison to the control group (Fig. 5).

Due to differing reports on the effect of gender, FFA and derivatives were also compared by aggregating the study group according to gender (Supplementary Tables S1, S2). For the medium chain fatty acids C13:0 tridecanoic acid, C14:1 myristolenic acid, C15:1 Cis-10-pentadecanoid acid, C16:0 palmitic acid, C16:1 palmitoleic acid, C17:1 Cis-10- heptadecanoid acid, C18:0 stearic acid, and C18:1n9 ct oleic acid, differences were statistically significant among groups (Supplementary Table S1). No significant gender differences were found in the level of inflammatory mediators (Supplementary Table S2).

## Discussion

Cerebral circulation is strictly regulated by vasoactive substances. There is a delicate balance between vasoconstrictor and vasodilating factors. During ischemia stroke, the autoregulation of cerebral circulation is handicapped, causing hyperaemia or hypoperfusion that disrupts the flow of blood. This situation contributes to the death of neuron cells due to incorrect blood flow. Traditional vasoactive mediators, such as nitric oxide and the peptide bound to calcitonin gene, were documented in order to ensure vasodilatation and for neuroprotection in the ischemic brain. The recurring domain is the identification of FFA and their derivatives which, as extracellular signalling particles, may lead to vasodilatation, probably also causing a neuroprotective effect^45,46^. Neuroprotection as an approach to brain therapy has been the subject of numerous discussions, fuelled by the necessity to improve the care of patients with acute stroke and by the identification of new medicines.

When analyzing the FFA profile in the plasma of ischemic stroke patients, it was observed that medium chain acids, such as C13:0 Tridecanoic acid, elongate into C16: 0 Palmitic acid. Additionally, the supply with the diet may also increase the amount of this acid in the plasma, resulting in a substrate in the course of further cascade^46,47^. Subsequently, C16:0 palmitic acid is desaturated into palmitoleic acid (C16:1) and elongated into stearic acid (C18:0), which is desaturated into its metabolites^48^. The pathway of this synthesis with the participation of SREBP-1c is stimulated by the high concentration of insulin and cholesterol. This is why the influence of the diet seems to be one of the key elements modulating the course of the reaction^49^.

**Figure 6:**
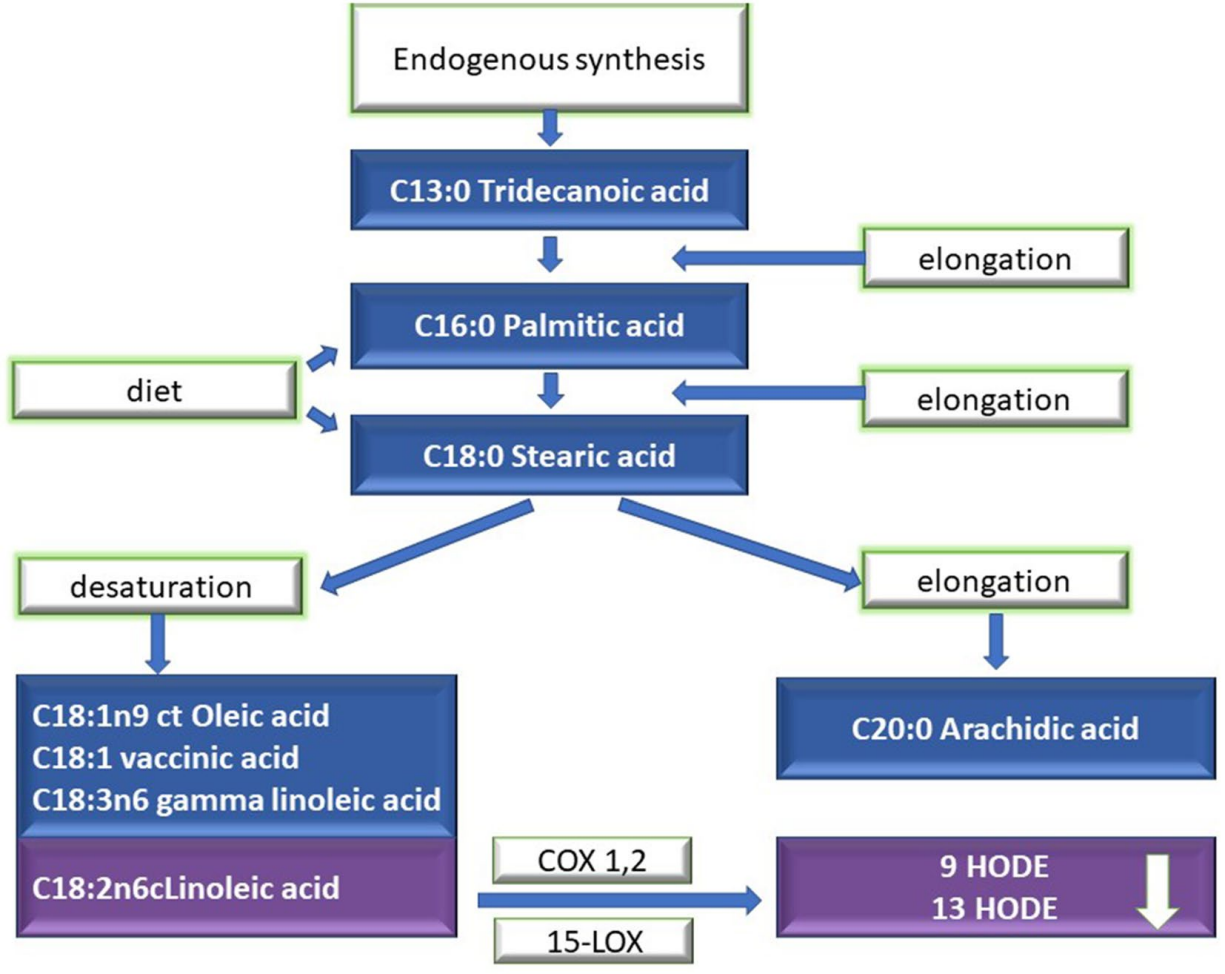
The synthesis of palmitic acid derivatives and proinflammatory mediators 9 and 13 HODE in ischemic stroke. *COX* cyclooxygenase, *CYP* cytochrom P450, *DGLA* dihomo-γ-linolenic acid, *EETs* epoxyeicosatetraenoic acids, *GLA* γ-linolenic acid, *HETEs* hydroxyeicosatetraenoic acids, *HODEs* hydroxyoctadecadienoic acids, *LOX* lipoxygenase, *LT* leucotrienes, *LX* lipoxins, *MaR1* maresins, *PG* prostaglandins, Rev(E1,D1)—resolvins, *TX* thromboxane.

One of the key metabolites is oleic acid, as presented in Fig. 6. Higher concentrations of oleic acid (C18:1w9) may be the cause of the growth and accumulation of lipids, supporting the occurrence of atherosclerosis and cardiovascular issues^50^. LA is the most widely present fatty acid in atherosclerotic plaques. These findings should not be extrapolated to dietary oleic acid intake because numerous epidemiological and intervention studies confirmed this observation, pointing to a strong relationship between the Mediterranean diet and circulatory system diseases. In this context, it seems that extra virgin olive oil (EVOO), the most representative element of this diet and a source of oleic acid, is important for the reduction of the frequency of occurrence of cardiovascular events, including myocardial infarction and stroke^51^. Diets providing increased amounts of LA can increase the synthesis of 13-HODE in the vascular endothelium, decreasing the adhesion of platelets and thrombogenicity. Another synthesized acid is C18:2n6 linoleic acid, which is the precursor of the synthesis of proinflammatory mediators 9 and 13 HODE, while gamma-linolenic acid (C18:3n-6) is the precursor of subsequent chains from the ɷ-6 family—eicosatrienoic acid (C20: 3n6) and arachidonic acid (C20:4n6). In this study, we observed an increase of all of these derivatives, except C18:2n6 linoleic acid which, while becoming a substrate for the synthesis of proinflammatory mediators 9 and 13 HODE, was partially used up. The lower level of these mediators in the IS group than in the control group means that the synthesis of both mediators is insufficient in the inflammatory reaction associated with ischemic stroke. Hydroxyoctadecadienoic acids (HODEs) are stable oxidation products, the generation of which is increased when oxidative stress is increased, such as in diabetes^52^. The influence of these derivatives has not been studied yet with reference to ischemic stroke. However, it was observed that in prior atherosclerosis, 13-HODE is created in macrophages through lipoxygenase-1, which increases protective mechanisms and leads to the increased removal of lipids from cells loaded with lipids from the arterial wall. Therefore, the activation of peroxisome proliferator-activated receptor (PPAR) is initiated and in later atherosclerosis both 9-HODE and 13-HODE are formed non-enzymatically^53^. Fatty acids and eicosanoids are activated PPAR nuclear transcription factors. PPAR-γ2 is the predominant form in adipose tissue. 13-HODE, created through the activity of 15-LOX-1, can also increase the reverse transportation of cholesterol through a mechanism encompassing PPAR-α^54^. Although the increased expression of 15-LOX-1 is a quality of early atherosclerosis, the activity of 13-HODE may in fact have a protective effect at this stage of the disease. However, at a later stage, both 9 and 13 HODE increase the susceptibility of vascular cells to apoptosis, which may contribute to the formation of atherosclerotic plaque^55^. Moreover, vascular smooth muscle cells (VSMC) may be stimulated by HODE generated by neighbouring cells, including platelets and macrophages. Limor et al. observed that vascular smooth muscle cells express 12/15-LOX, and hence are capable of synthesizing 12-HETE, 15-HETE, and 13-HODE^56^.

In the ischemic stroke environment, in which the flow of cerebral blood is obstructed, brain tissue that lacks oxygen initiates the separation of arachidonic acid (AA) from the double-layer phospholipid membrane. After release, AA undergoes both oxidative metabolism independent of enzymes and mediated by enzymes such as lipoxygenase and cyclooxygenase. As a result of the amplified synthesis reactions, numerous biologically active metabolites occur, which contribute to the prognosis of the pathological stroke^47^.

When analyzing further elongation and increases in the synthesis of arachidonic acid and the cascade of its transformations, which enable the synthesis of prostaglandins (PG), thromboxanes (TX) series 2, leukotrienes (LT) series 4, and 5, 12, 15 HETE, we observed a significant amplification of inflammation in the levels of selected mediators. HETE derivatives are strongly pro-inflammatory and stimulate clotting, atherosclerotic changes, inflammatory and allergic reactions, the proliferation of cells and malignant tissue growth^57^. In our study, we observed the activation of three synthesis pathways from the cascade of arachidonic acid:

- COX-1,2—leading to the synthesis of PGE2,
- LOX-15—leading to the synthesis of 15 HETE,
- LOX-5—leading to the synthesis of leukotrienes.

**Figure 7:**
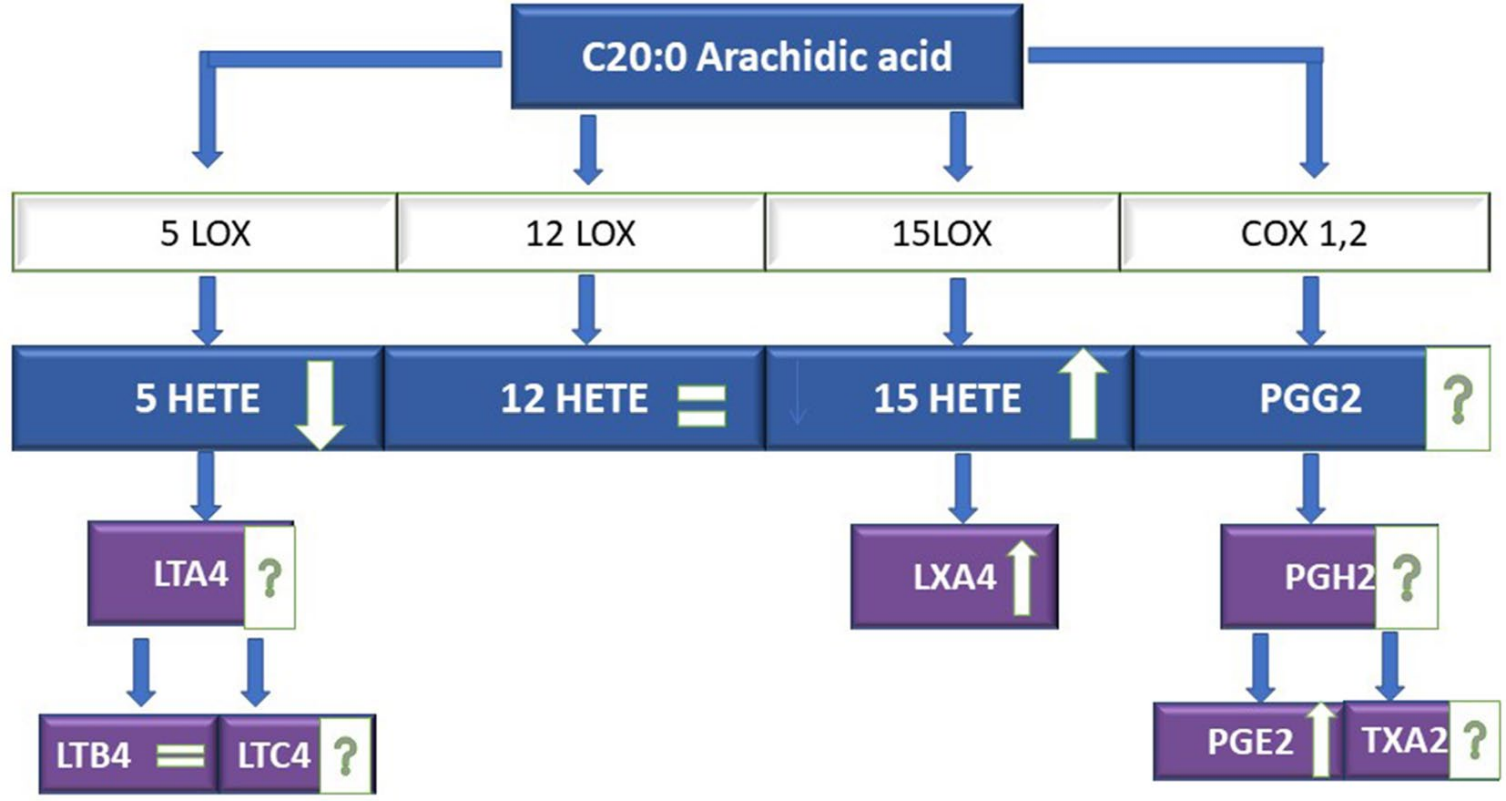
The synthesis of arachidonic acid derivatives and proinflammatory mediators in ischemic stroke. *COX* cyclooxygenase, *CYP* cytochrom P450, *DGLA* dihomo-γ-linolenic acid, *EETs* epoxyeicosatetraenoic acids, *GLA* γ-linolenic acid, *HETEs* hydroxyeicosatetraenoic acids, *HODEs* hydroxyoctadecadienoic acids, *LOX* lipoxygenase, *LT* leucotrienes, *LX* lipoxins, *MaR1* maresins, *PG* prostaglandins, Rev(E1,D1)—resolvins, *TX* thromboxane.

Here, the LOX-5 pathway appears to have been activated and its metabolites were partially used up. However, we cannot be absolutely sure because we did not study the entire leukotrienes family (LTC4, LTD4, LTE4). The outline of this part of our discussion is presented in Fig. 7.

The pathways of lipoxin synthesis, which are specialised pro-resolving mediators (SPMs), were not activated. Therefore, particles separating inflammation could be used in the early treatment stages of ischemic stroke. Analogues like BML-111 have extended biological activity and can, therefore, be a better therapeutic option (synthetic LXA4 analogue also called heptanoate 5 (S), 6 (R)-7-trihydroxymethyl)^58,59^. Due to the fact that 12/15-lipoxygenase (12/15-LOX—the key enzymes of arachidonic acid cascade) contribute to both neural cell death and damage, the inhibition of 12/15-LOX can ensure the multifactor protection against ischemic issues, as shown by our study and the studies of other authors^60^.

**Figure 8:**
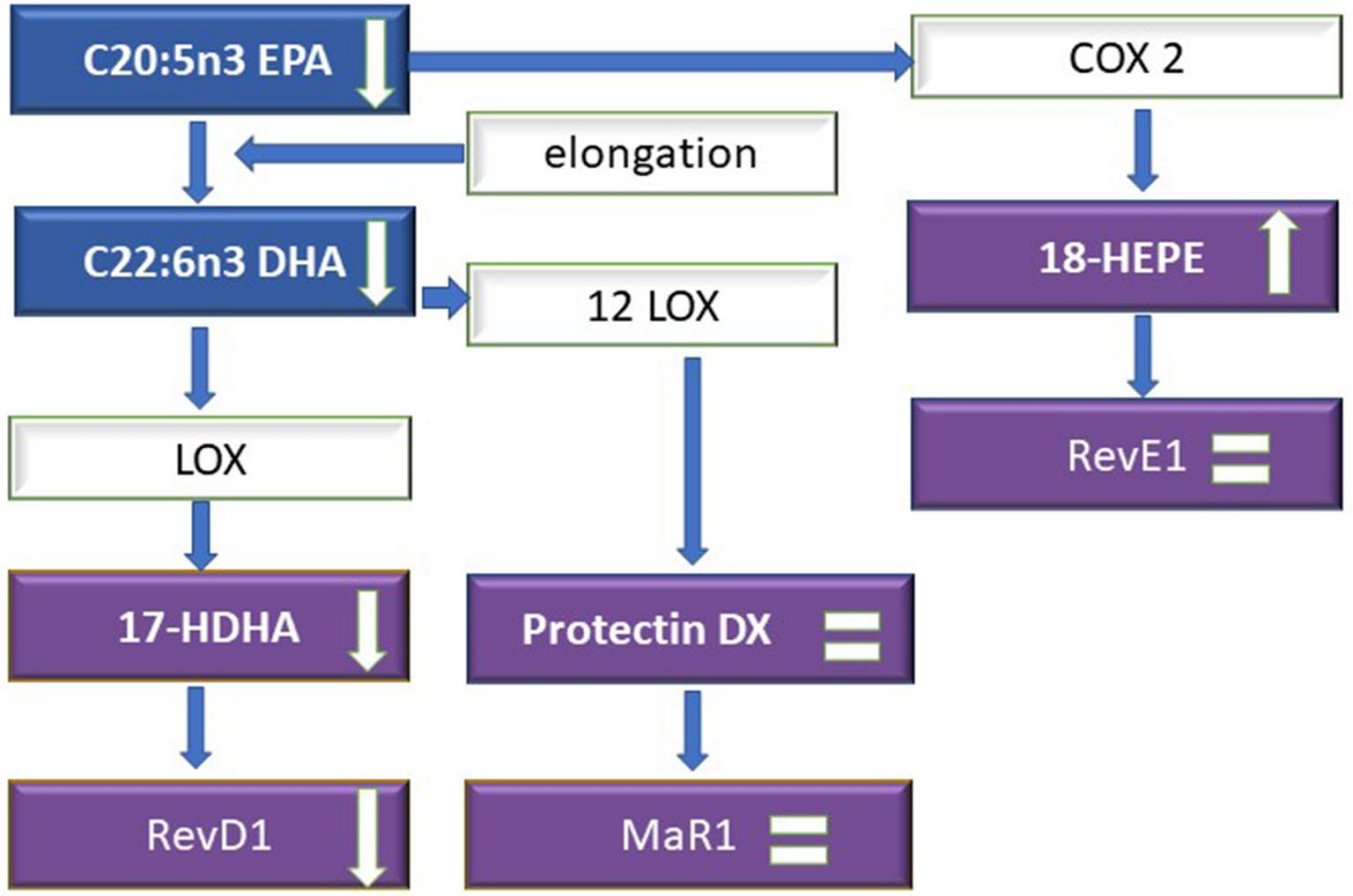
The synthesis of omega-3 fatty acid derivatives (EPA, DHA) in ischemic stroke. *COX* cyclooxygenase, *CYP* cytochrom P450, *DGLA* dihomo-γ-linolenic acid, *EETs* epoxyeicosatetraenoic acids, *GLA* γ-linolenic acid, *HETEs* hydroxyeicosatetraenoic acids, *HODEs* hydroxyoctadecadienoic acids, *LOX* lipoxygenase, *LT* leucotrienes, *LX* lipoxins, *MaR1* maresins, *PG* prostaglandins, Rev(E1,D1)—resolvins, *TX* thromboxane.

When performing the analysis of long chain fatty acids of the omega-3 family, it was observed that they are involved at this stage of ischemic stroke, especially DHA-derivative resolvins. However, the synthesis through cyclooxygenase proceeds to a lesser, yet still significant, extent. In the case of EPA and DHA derivatives, we did not observe the involvement of all pathways responsible for the quenching of inflammation. This relationship was not observed with reference to the synthesis of maresins through 12LOX, as presented in Fig. 8. However, maresins may also serve as therapeutic alternatives during the later stages of treatment, particularly due to the fact that the 7S-MaR1 analogue accelerates and improves the clearance of neutrophils and reduces the accumulation of macrophages in the damaged spinal cord^61^. Further studies of this subject are necessary.

A limitation in our study is the fact that we did not account for the APOE-ε4 of hosts who, due to increased insulin resistance markers, are more susceptible to both the positive and negative biological effects of fatty acids in response to dietary changes^62–64^. Another limitation is the unequal gender distribution in the control group relative to stroke patients, although no differences were found in the level of inflammatory mediators by gender.

## Conclusions

Ischemic stroke is associated with the presence of a strong inflammatory reaction, in which arachidonic acid plays a key role. Derivatives are synthesized using three pathways—5LOX, 15LOX and COX1,2—with the participation of prostaglandins; it seems that 12LOX is not involved in the process. The pathways leading to the synthesis of EPA derivatives, particularly DHA, are also intensified, leading to the increased consumption of resolvins, which are DHA derivatives. Furthermore, the pathway of palmitic acid derivatives (9 and 13 HODE) is also activated, though it seems to play a lesser role. The potential to affect and accelerate the stage of inflammation quenching after stroke seems to be a promising strategy in the treatment of stroke in its early stage. Our study points to lipoxins, RevD1 and HODE as the most important derivatives for this task.

## Supplementary information

Supplementary Information
