# Supplementary material for: Lipoxins, RevD1 and 9, 13 HODE as the most important derivatives after an early incident of ischemic stroke
Source: Sci Rep. 2020 Jul 30;10:12849. doi: 10.1038/s41598-020-69831-0 (PMC7393087; doi:10.1038/s41598-020-69831-0)
Supplement: Supplementary file 1 — Supplementary Information [file 41598_2020_69831_MOESM1_ESM.docx]

**Lipoxins, RevD1 and 9, 13 HODE as the most important derivatives after an early incident of ischemic stroke**

Małgorzata Szczuko^1^, Dariusz Kotlęga^2,3^, Joanna Palma^1^, Agnieszka Zembroń-Łacny^4^, Anna Tylutka^4^, Monika Gołąb-Janowska^2^, Arleta Drozd^1^

Tab. 1 Comparison of FFA participation in ischemic stroke by gender [%]

| **FFA [%]** | **Men**  **mean± SD** | **Women**  **mean± SD** | **p-value** |
| --- | --- | --- | --- |
| **C13:0 Tridecanoic acid** | 0.333 ± 0.090 | 0.287 ± 0.088 | **0.0324** |
| **C14:0 Myristic acid** | 1.197 ± 0.429 | 1.216 ± 0.345 | 0.8265 |
| **C14:1 Myristolenic acid** | 0.060 ± 0.035 | 0.077 ± 0.038 | **0.0498** |
| **C15:0 Pentadecanoid acid** | 0.224 ± 0.123 | 0.212 ± 0.095 | 0.6306 |
| **C15:1 Cis-10-Pentadecanoid acid** | 0.093 ± 0.038 | 0.072 ± 0.032 | **0.0134** |
| **C16:0 Palmitic acid** | 27.280 ± 1.405 | 26.426 ± 1.899 | **0.0341** |
| **C16:1 Palmitoleic acid** | 1.825 ± 0.530 | 2.377 ± 0.806 | **0.0011** |
| **C17:0 Heptadecanoic acid** | 0.298 ± 0.043 | 0.305 ± 0.056 | 0.5655 |
| **C17:1 Cis-10- Heptadecanoid acid** | 0.101 ± 0.035 | 0.083 ± 0.033 | **0.0212** |
| **C18:0 Stearic acid** | 13.860 ± 1.719 | 12.884 ± 2.089 | **0.0334** |
| **C18:1n9 ct Oleic acid** | 21.577 ± 2.879 | 23.388 ± 4.116 | **0.0351** |
| **C18:1 Vaccinic acid** | 1.920 ± 0.325 | 2.025 ± 0.366 | 0.2016 |
| **C18:2n6c Linoleic acid** | 11,874 ± 2.284 | 11.275 ± 2.364 | 0.2725 |
| **C18:2n6t Linoleic acid** | 6.075 ± 1.986 | 6.193 ± 1.909 | 0.7959 |
| **C18:3n6 Gamma linoleic acid** | 0.331 ± 0.159 | 0.430 ± 0.206 | **0.0252** |
| **C18:3n3 Linolenic acid** | 0.500 ± 0.140 | 0.507 ± 0.173 | 0.8620 |
| **C18:4 Stearidonic acid** | 0.050 ± 0.021 | 0.064 ± 0.030 | **0.0283** |
| **C20:0 Arachidic acid** | 0.199 ± 0.041 | 0.212 ± 0.091 | 0.4313 |
| **C22:1/C20:1 cis11- Eicosanic acid** | 0.168 ± 0.035 | 0.187 ± 0.086 | 0.2381 |
| **C20:2 cis-11-Eicodienoic acid** | 0.149 ± 0.029 | 0.152 ± 0.038 | 0.7977 |
| **C20:3n6 Eicosatrienoic acid** | 1.250 ± 0.269 | 1.308 ± 0.338 | 0.4288 |
| **C20:4n6 Arachidonic acid** | 6.470 ± 1.391 | 6.176 ± 1.252 | 0.3408 |
| **C20:3n3 Cis-11-Eicosatrienoic acid** | 0.033 ± 0.016 | 0.029 ± 0.013 | 0.2527 |
| **C20:5n3 EPA** | 0.582 ± 0.295 | 0.620 ± 0.228 | 0.5276 |
| **C22:0 Behenic acid** | 0.217 ± 0.090 | 0.232 ± 0.105 | 0.5313 |
| **C22:1n9 13 (Erucic acid** | 0.071 ± 0.017 | 0014 ± 0.014 | 0.1603 |
| **C22:2 Cis-Docodienoic acid** | 0.019 ± 0.038 | 0.015 ± 0.007 | 0.1160 |
| **C23:0 Tricosanoic acid** | 0.239 ± 0.013 | 0.228 ± 0.009 | 0.7693 |
| **C22:4n6 Docosatetraenoic acid** | 0.231 ± 0.147 | 0.216 ± 0.157 | 0.5894 |
| **C22:5w3 Docosapentaenoic acid** | 0.475 ± 0.131 | 0.448 ± 0.105 | 0.6188 |
| **C24:0 Lignoceric acid** | 0.150 ± 0.334 | 0.155 ± 0.095 | 0.7646 |
| **C22:6n3 DHA** | 1.773 ± 0.072 | 1.736 ± 0.080 | 0.7657 |
| **C24:1 Nervonic acid** | 0.377 ± 0.575 | 0.418 ± 0.499 | 0.4801 |

Text in bold (p -value)- statistically significant

Tab. 2 Comparing the level of inflammatory mediators in terms of gender [ug/mL].

| **Inflammation mediators** | **Men**  **mean± SD** | **Women**  **mean± SD** | **p-value*** |
| --- | --- | --- | --- |
| **RevE1** | 0.055 ± 0.079 | 0.066 ± 0.104 | 0.620 |
| **Prostaglandin E2** | 3.088 ± 3.069 | 3.760 ± 5.330 | 0.521 |
| **RevD1** | 0.213 ± 0.337 | 0.148 ± 0.163 | 0.270 |
| **LTX A4 5S, 6R** | 0.038 ± 0.221 | 0.000 ± 0.000 | 0.262 |
| **LTX A4 5S, 6R, 15R** | 0.021 ± 0.035 | 0.026 ± 0.047 | 0.625 |
| **Protectin DX** | 0.042 ± 0.048 | 0.051 ± 0.074 | 0.555 |
| **MaR 1** | 0.032 ± 0.012 | 0.032 ± 0.019 | 0.978 |
| **Leukotriene B4** | 0.026 ± 0.013 | 0.027 ± 0.015 | 0.877 |
| **18-HEPE** | 0.108 ± 0.040 | 0.111 ± 0.035 | 0.736 |
| **16-HETE** | 0.000 ± 0.000 | 0.018 ± 0.089 | 0.250 |
| **13-HODE** | 0.035 ± 0.038 | 0.030 ± 0.20 | 0.407 |
| **9-HODE** | 0.036 ± 0.036 | 0.031 ± 0.019 | 0.418 |
| **15-HETE** | 0.269 ± 0.0168 | 0.312 ± 0.227 | 0.364 |
| **17-HDHA** | 0.112 ± 0.083 | 0.129 ± 0.086 | 0.381 |
| **12-HETE** | 1.810 ± 1.134 | 1.745 ± 1.133 | 0.807 |
| **5 oxo ETE** | 0.183 ± 0.0121 | 0.184 ± 0.086 | 0.994 |
| **5-HETE** | 0.022 ± 0.009 | 0.027 ± 0.015 | 0.094 |

*No statistically significant differences
